# Electromagnetic navigation system for acetabular component placement in total hip arthroplasty is more precise and accurate than the freehand technique: a randomized, controlled trial with 84 patients

**DOI:** 10.1080/17453674.2020.1783073

**Published:** 2020-07-01

**Authors:** Rene Mihalič, Jurij Zdovc, Janez Mohar, Rihard Trebše

**Affiliations:** a Valdoltra Orthopaedic Hospital, Ankaran; b University of Ljubljana, Faculty of Pharmacy, Ljubljana; c University of Ljubljana, Faculty of Medicine, Ljubljana, Slovenia

## Abstract

Background and purpose — The accuracy of conventional navigation systems depends on precise registration of bony landmarks. We investigated the clinical use of electromagnetic navigation (EMN), with a unique device for precise determination of the anterior pelvic plane.

Patients and methods — We randomly allocated patients scheduled for total hip arthroplasty into 2 groups of 42 patients each. In the study group, cups were placed at the predetermined target angles (inclination: 42.5°; anteversion: 15°) with the support of EMN. In the control group, cups were placed freehand aiming at the same target angles. Postoperatively the true position of the cup was determined using computed tomography scan of the pelvis. Precision (root mean squared error, RMSE) bias (mean bias error, ME), accuracy, and duration of surgery were compared between the methods.

Results — Cup anteversion was more accurate and precise in the navigated group. The ME in the navigated and freehand group was –1.7° (95% CI –2.4 to 1.1) and –4.5° (CI –6.5 to 2.5), respectively. The RMSE in the navigated and freehand group was 2.8° (CI 2.3–3.2) and 8.0° (CI 6.3–9.5), respectively. The inclination was also more precise in the navigated group, with the RMSE in the navigated and freehand group at 4.6° (CI 3.4–5.9) and 6.5° (CI 5.4–7.5), respectively. The accuracy of the inclination and the duration of surgeries were similar between the groups.

Interpretation — Cup placement with the help of EMN is more precise than the freehand technique and it does not affect the duration of surgery.

Optimal cup placement is crucial to the success of total hip arthroplasty (THA) since it is associated with lower rates of dislocation, prolonged implant survival, and better quality of life of the patient (Learmonth et al. 2007). For cup position in THA the safe zone was defined by Lewinnek et al. (1978), with recommended inclination and anteversion angles of 40° ± 10° and 15° ± 10°, respectively. Several studies have demonstrated that using the freehand technique for cup placement within the safe zone remains a challenge even for high-volume surgeons. More than 75% of cups are still inadvertently placed out of the safe zone (Digioia et al. 2002, Saxler et al. 2004, Bosker et al. 2007). Several studies have reported that the cup placement could be optimized using imageless navigation, which is a more reproducible technique compared with a freehand THA (Digioia et al. 2002, Kalteis et al. 2006a, Hohmann et al. 2011, Lass et al. 2014). Those studies were mostly performed with different producers’ stereo-optical navigation systems with the same basic concept (Renkawitz et al. 2009). To assure the accuracy of such a system, the tracker must be large, and therefore placed outside the surgical incision. This is related to additional morbidity (Dorr et al. 2005, Kamara et al. 2017). The accuracy depends on precise registration of bony landmarks (Dorr et al. 2005, Lass et al. 2014), which are necessary for the determination of the reference plane. The registration of the reference points is mostly affected by the thickness of the overlying soft tissues. This can tilt the virtual reference plane and contribute to the systemic error (Hohmann et al. 2011), especially in obese patients (Parratte and Argenson 2007, Wassilew et al. 2012, Buller et al. 2019). To avoid imprecise reference plane determination, a different imageless navigation concept was introduced. This system consists of an electromagnetic transmitter and sensors, which are placed inside the incision and on instruments. Additionally, we developed a particular tool (Navi-frame) to overcome the difficulties in the registration of bony landmarks for correct anterior pelvic plane (APP) determination. The basic idea was that at least 3 non-collinear points describe a plane. In THA these 3 points are represented by the two anterior superior iliac spines (ASIS) and the pubic tubercle. The real APP is registered with the placement of the Navi-frame on these 3 points (Figure 1). This presents a major improvement compared with the stereo-optical systems.

Our 2 hypotheses for this study were:

1. An electromagnetic navigation (EMN) system enables more accurate and precise cup placement in THA than the freehand technique, regardless of the patient’s BMI.
2. The EMN system does not affect the duration of surgery.

## Patients and methods

### Study design and patient selection

Before the study design, we performed a pilot study (part of the validation process of the EMN system), including 10 patients in each group (navigated and freehand group), which was a basis for power analysis and also represented a learning period for handling the EMN system.

This was a randomized, controlled clinical trial of 2 groups of 42 patients, all scheduled for THA between May 4, 2017 and February 2, 2018. The patient data included: diagnosis, age, sex, BMI, Harris Hip Score (HHS), and side. In the study group (EHIP), patients underwent the EMN-assisted cup placement during THA. In the control group (freehand), patients underwent conventional freehand cup placement. The inclusion criteria were age above 18 years, unilateral surgery, osteoarthritis of the hip, no previous surgery on the affected hip, implantation of the same acetabular component through the same approach, and signed informed consent. 3 high-volume surgeons performed all procedures. We followed the CONSORT guidelines. Within the cohort of 137 consecutive patients scheduled for primary THA, 84 patients met the inclusion criteria and were randomly allocated in a 1:1 ratio into the EHIP group and the control (freehand) group (Figure 2). Randomization was conducted using computer-generated numbers from the Research Randomizer System. Even numbers represented the EHIP group.

**Figure 1.**
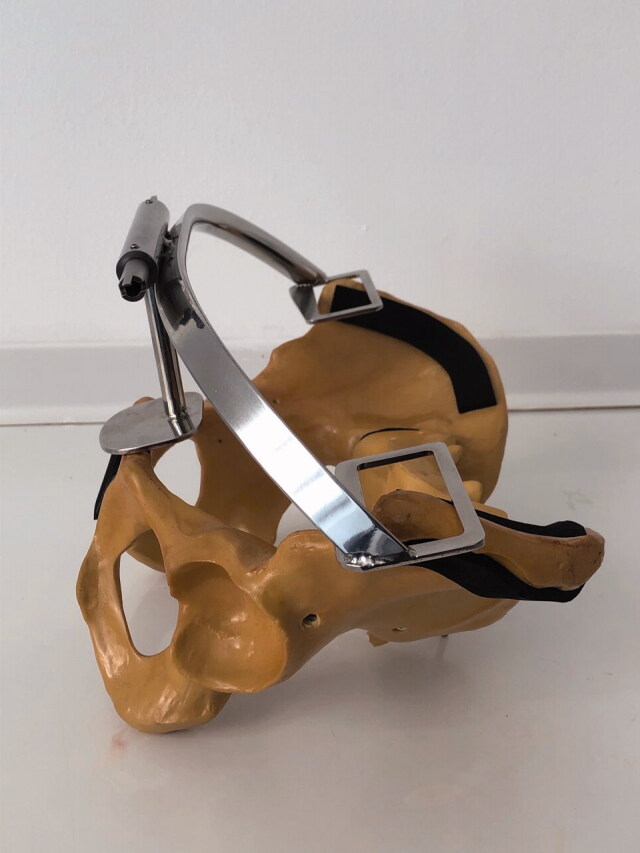
Position of the Navi-frame on the sawbones model of the pelvis.

**Figure 2.**
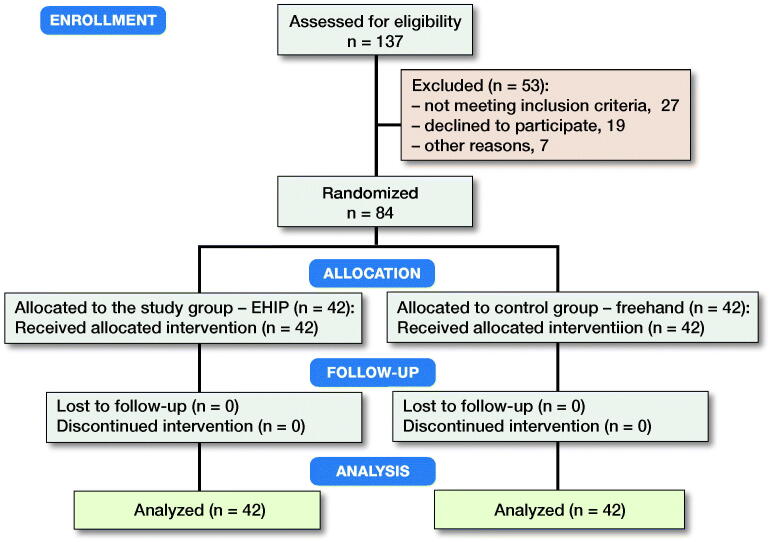
CONSORT flow diagram of the study.

### Surgical procedure and postoperative evaluations

**Figure 3.**
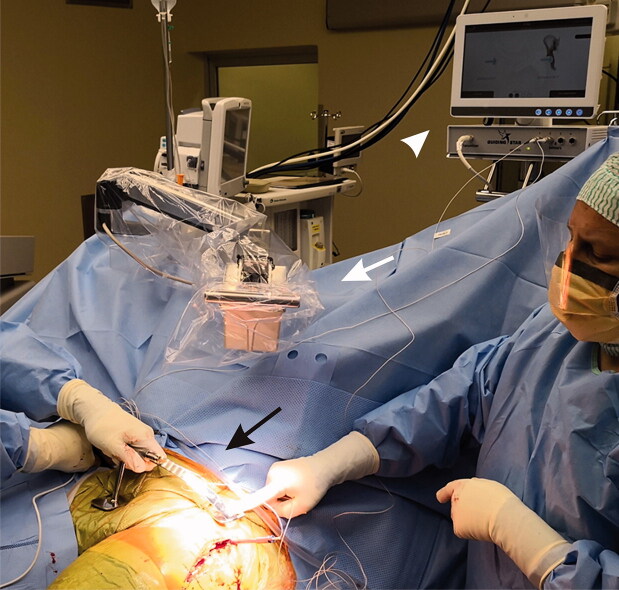
Determination of the anterior pelvic plane with Navi-frame (black arrow) and navigation system consisting of electromagnetic transmitter (white arrow) and monitor (white arrowhead).

**Figure 4.**
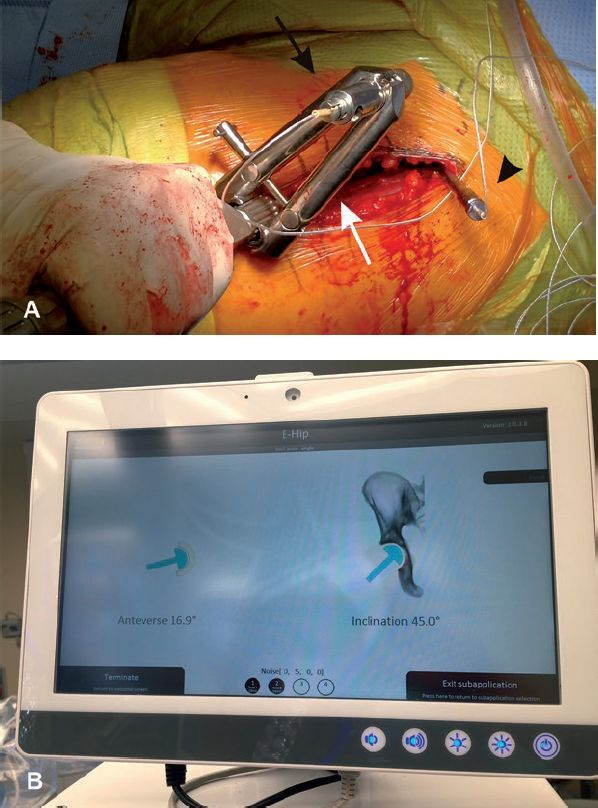
A. Implantation of the acetabular component with conventional cup applicator equipped with holder (white arrow) for measuring sensor (black arrow). Reference sensor (black arrowhead). B. Monitor with real-time information on acetabular component position.

A modified Hardinge approach in the supine position was used and a cementless cup and stem from the same manufacturer were implanted in all cases (Allofit/Alloclassic Zimmer Biomet, Warsaw, IN, USA). In the EHIP group, during patient draping a sterilely covered arm with the electromagnetic transmitter of the EMN system (Guiding Star, E-Hip module, Ekliptik d.o.o., Ljubljana, Slovenia) was mounted on the operating table and connected to the central unit equipped with a monitor. No additional preoperative time was needed to prepare the EMN system. After resection of the femoral head, a specially designed Steinmann pin (diameter 4.5 mm) with a reference sensor on it was mounted above the acetabular edge, without additional skin and soft tissue dissection. APP was then determined with the help of the Navi-frame equipped with the measuring sensor. The Navi-frame was place-pressed on both ASIS and the pubic tubercle. When the position of the frame was correct, the APP was registered (Figure 3). After acetabular preparation, the cup was impacted with the help of a conventional cup holder with a measuring sensor on it aiming to place the cup around predefined target angles (42.5° for inclination and 15° for anteversion). The values of both angles were displayed on the monitor and registered by the EMN system. Those values represented the basis for later calculations of accuracy and precision of the EMN system (Figure 4). All manipulations of the navigation system were performed after the skin incision and represent part of the measured surgical duration (skin incision to last skin suture).

In the freehand group, the cup was placed freehand, aiming to place it inside the predefined target angles, with the help of visible anatomical landmarks around the acetabulum and relying on the surgeon’s ability to estimate the patient’s real position on the operating table. Predefined target angles represented the basis for later calculations of precision and accuracy of the freehand technique.

**Figure 5.**
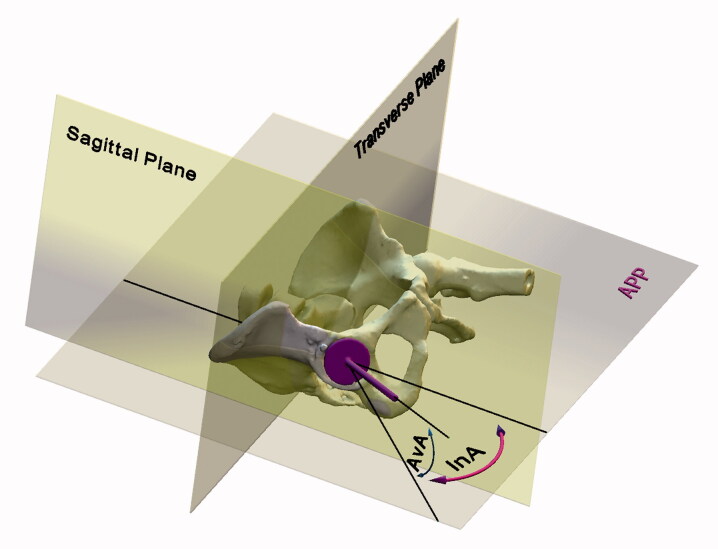
Postoperative measurements of cup position (APP = anterior pelvic plane).

Postoperatively (up to 48 hours after surgery) all patients underwent CT scans of hip and pelvis for the determination of the actual acetabular component position, which served as a reference (Kalteis et al. 2006b, Lass et al. 2014). The position of the pelvis was standardized by reformatting the images to the APP. Single measurements of the inclination and anteversion angles based on the CT scans of hips and pelvises were made by the independent technician, with the help of special CAD/CAM (computer-aided design/manufacturing) software (EBS software, Ekliptik d.o.o., Ljubljana, Slovenia), where the technician defined the APP, the sagittal plane, the transverse plane, and the axis of the cup (line perpendicular to the plane defined by the outer circumference of the cup), and the measurements occurred automatically based on the combined algorithm by Murray (1993), and by Hohmann et al. (2011) (Figure 5). All patients underwent the same perioperative antibiotic prophylaxis, pain management, and rehabilitation protocols.

### Statistics

The sample size was calculated with the G*Power software tool (http://www.gpower.hhu.de/) based on the preliminary data obtained from the validation of the navigation system (Mihalic and Trebse 2016). Study power (1–β) was set to 0.8 and the significance level (α) to 0.05. Descriptive statistics were given as mean and range for continuous variables and as percentages for categorical variables. Continuous variables for both groups were compared using a 2-tailed Student’s independent t-test, and categorical variables were compared with the chi-square test.

For the comparison of method performance in EHIP and the freehand group, the target angles during the THA and angles measured on the postoperative CT scan (true angles) were used to calculate the deviation from the target angle for every patient. Based on those deviations, the method bias, precision, and accuracy were determined. Bias was expressed as the mean bias error (ME), which was calculated as the average difference between the target angles and the true angle. The negative difference between the target and true angle is interpreted as the method’s underprediction of the true angle. Precision was expressed as the root mean squared error (RMSE), which was calculated as the standard deviation of the differences between the target angle and the true angle. ME and RMSE for the inclination and anteversion angles were primary outcomes of our study. Accuracy was represented by a combination of ME and RMSE (Sheiner and Beal 1981). The lower absolute value of ME and the lower RMSE represent less method bias and better precision, respectively, and subsequently better method accuracy. The duration of the surgery was the secondary outcome. R software, version 3.6.0 (R Development Core Team, Vienna, Austria) and package boot (http://www.R-project.org) was used to perform bootstrapping with replacement method (number of virtual samples = 10,000) and to obtain the 95% confidence intervals (CI) for the ME and RMSE. Other statistical analyses were performed using the Statistical Package for the Social Sciences (SPSS), version 25.0 (IBM Corp, Armonk, NY, USA). P < 0.05 was considered significant.

### Ethics, registration, funding, and potential conflicts of interest

The study design was approved by the National Committee of Medical Ethics (77/05/12) and was performed in agreement with the Helsinki II declaration. The study was registered at ClinicalTrials.gov (NCT04101864). This research did not receive any specific grant from funding agencies in the public, commercial, or not-for-profit sectors. Regarding this study the authors have no conflicts of interest.

## Results

### Demographic data

The demographic characteristics of both groups were similar (Table 1).

**Table 1.** Demographic data. Values are mean (standard deviation) unless otherwise specified

	Type of operation	
	Freehand	EHIP
Factor	(n = 42)	(n = 42)
Sex: female/male, n	22/20	21/21
Age	66 (11)	67 (11)
Body mass index	29 (5.7)	30 (4.3)
HSS, preoperative	62 (17)	56 (17)

EHIP, electromagnetic navigation surgery.

HHS, Harris Hip Score

### Surgical duration

The average duration of surgeries was similar between the groups: in the EHIP group 70 minutes (SD 10) and 70 minutes (SD 13) in the freehand group.

### Radiographic parameters

**Figure 6.**
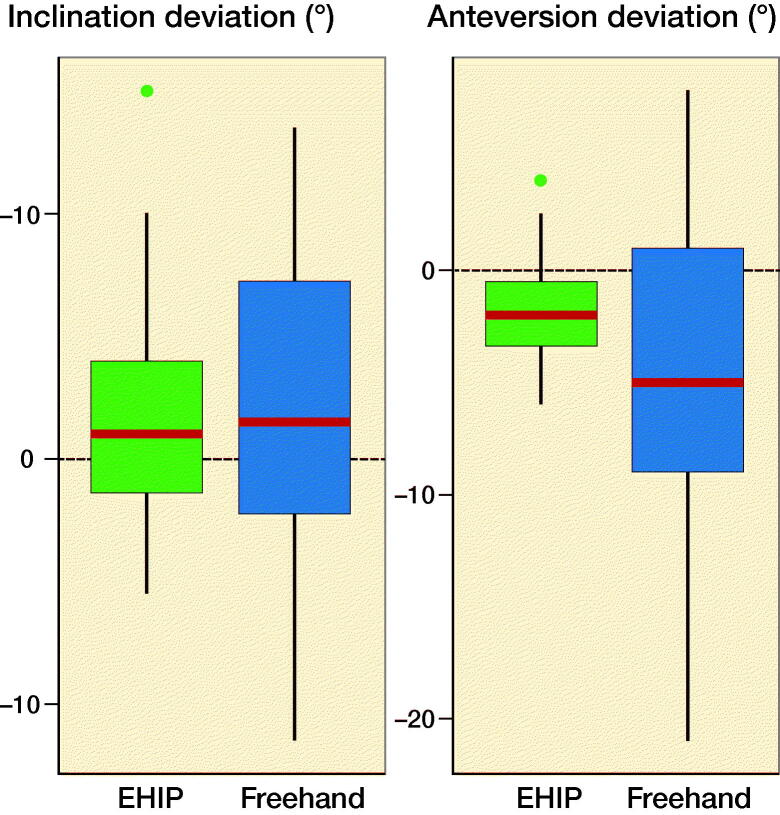
Deviations of the inclination angle measurements (left panel) and anteversion angle measurements (right panel) in the EHIP (study group) and the freehand group. Median (red line across boxes), 1st and 3rd quartile (lower and upper hinges), minimum and maximum non outlying (< 1.5 times interquartile range) values (whiskers).

**Figure 7.**
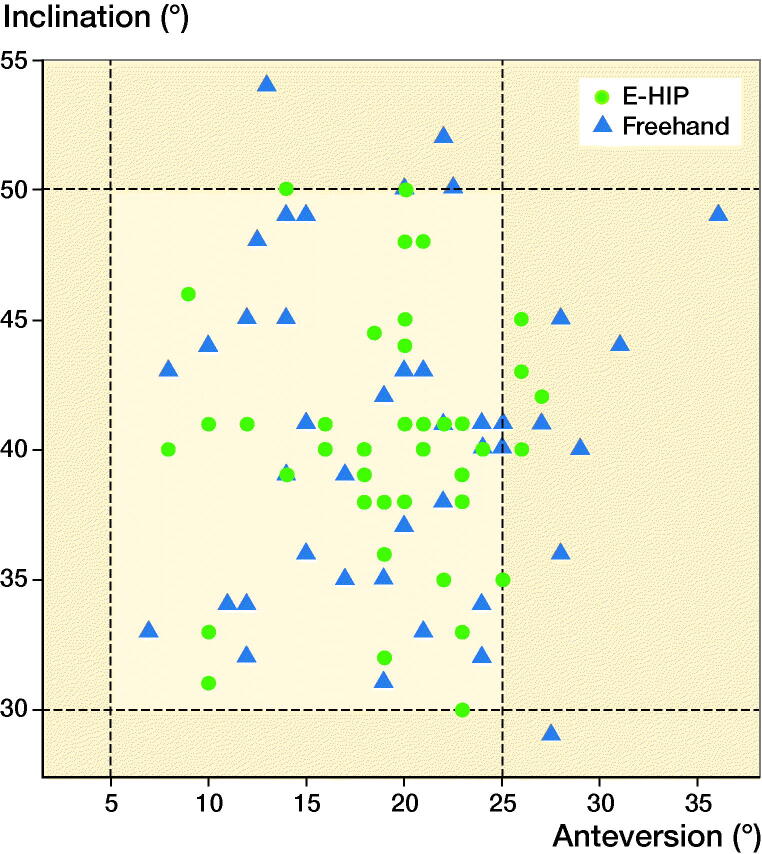
Distribution of cup positions for both groups. Area between 30° and 50° for inclination and between 5° and 25° represents Lewinnek safe zone.

For both angles, the observed range of deviations was lower in the EHIP group (Figure 6). Precision presented by the RMSE for both angles was statistically significantly higher in the EHIP group (Table 2). Accuracy, presented by a combination of RMSE and ME, was statistically significantly higher for the anteversion angle in the EHIP group (Table 2). However, we did not observe any difference in method bias for the inclination angle when comparing the two groups (Table 2). Regarding Lewinnek’s safe zone there were 4 outliers in the EHIP, and 9 outliers in the freehand group (Figure 7).

**Table 2.** Comparison of accuracy and precision for inclination and anteversion angles. Values are mean (95% confidence interval)

	Type of operation		
	Freehand	EHIP	
Factor	(n = 42)	(n = 42)	p-value **^a^**
Inclination			
ME (°)	1.9 (0.0 to 3.8)	1.7 (0.4 to 3.0)	0.9
RMSE (°)	6.5 (5.4 to 7.5)	4.6 (3.4 to 5.9)	0.02
Anteversion			
ME (°)	–4.5 (–6.5 to –2.5)	–1.7 (–2.4 to –1.1)	0.01
RMSE (°)	8.0 (6.4 to 9.6)	2.8 (2.3 to 3.3)	< 0.001

**^a^** 2-tailed Student’s independent t-test, based on the bootstrap sample distributions.

EHIP, electromagnetic navigation surgery.

ME, mean bias error.

RMSE, root mean squared error.

Additionally, no significant association was observed between the precision and accuracy and BMI. The ME and RMSE were similar comparing non-obese patients with BMI ` 30 and obese patients with BMI ≥ 30 for both angles, and for both methods (Table 3).

**Table 3.** Comparison of accuracy and precision of inclination and anteversion angles for patients with body mass index (BMI) ≤ 30 and ≥ 30 in both groups. Values are mean (95% confidence interval)

Factor	BMI < 30	BMI ≥ 30	p-value **^a^**
Freehand	(n = 15	(n = 27	
Inclination			
ME (°)	3.0 (0.7 to 5.3)	–0.2 (–3.1 to 2.7)	0.1
RMSE (°)	6.8 (5.7 to 7.9)	5.7 (3.6 to 8.2)	0.4
Anteversion			
ME (°)	–5.0 (–7.3 to –2.6)	–3.6 (–7.2 to –0.1)	0.5
RMSE (°)	8.1 (6.0 to 10)	7.8 (5.4 to 10)	0.9
EHIP	(n = 22	(n = 20	
Inclination			
ME (°)	1.9 (0.1 to 3.7)	1.5 (–0.5 to 3.3)	0.8
RMSE (°)	4.7 (3.7 to 5.8)	4.6 (2.2 to 7.2)	0.9
Anteversion			
ME (°)	–1.8 (–2.8 to –0.9)	–1.6 (–2.5 to –0.7)	0.8
RMSE (°)	2.9 (2.3 to 3.5)	2.6 (1.9 to 3.4)	0.5

For footnotes, see Table 2.

## Discussion

Currently, there are still various opinions regarding the benefits of navigation and other computer-aided systems in THA. Many studies demonstrated that navigation is superior to freehand techniques regarding accuracy and precision of the cup placement (Dorr et al. 2005, Parratte and Argenson 2007, Najarian et al. 2009, Hohmann et al. 2011, Lass et al. 2014, Buller et al. 2019). Importantly, despite there being several advantages of the existing navigation systems (accuracy and precision) (Dorr et al. 2005, Parratte and Argenson 2007, Najarian et al. 2009, Hohmann et al. 2011, Lass et al. 2014, Buller et al. 2019), they also have considerable disadvantages including tracker-related harm (Dorr et al. 2005, Kamara et al. 2017), longer duration of surgery (Parratte and Argenson 2007, Najarian et al. 2009, Lass et al. 2014, Liu et al. 2015), and higher costs.

Our study confirmed the first hypothesis. The presented EMN system with the use of the Navi-frame provides more precise cup placements in THA compared with the freehand technique. Additionally, the EMN system demonstrated less bias in anteversion angle estimation, indicating better accuracy of cup placement for the anteversion angle. The observed accuracy and precision of the anteversion angle of the cup placement were below 3°. Additionally, we observed that accuracy and precision of navigated cup placement were unaffected by the patient’s BMI or thickness of the soft tissue overlaying the bony landmarks. The accuracy and the precision of navigated cup placement were similar comparing the obese (BMI ≥ 30) and the non-obese patients (BMI ≤ 30) (Table 3). We hypothesize this is due to the specially developed Navi-frame for APP determination, which captures all three bony landmarks at once and creates the most accurate approximation of real APP, regardless of the patient’s BMI.

In contrast to our observations, several studies have demonstrated the opposite: that the precision and accuracy of conventional imageless navigation systems is mostly affected by the soft tissue overlying the bony landmarks (Parratte and Argenson 2007, Parratte et al. 2007, Wassilew et al. 2012, Buller et al. 2019) and that the most important factor to avoid systemic error is precise registration of landmarks (Digioia et al. 2002, Parratte et al. 2008, Hohmann et al. 2011, Lass et al. 2014).

Paratte and Argenson (2007) concluded that the accuracy of acetabular component placement in obese patients is considerably affected by the soft tissue thickness over the bony landmarks, which probably affects the precision of registration of the APP and is the most obvious limitation factor of navigation systems currently on the market. The in vitro study by Paratte et al. (2008) evaluated the accuracy of percutaneous and ultrasound-based registration of bony landmarks for APP determination. They reported no statistically significant difference in terms of inclination. In contrast, anteversion errors were statistically significantly higher with percutaneous registration. Similar results were published by Wassilew et al. (2012), who compared the accuracy of an ultrasound-based navigation system and an imageless navigation system with surface registration. They also observed a statistically significant correlation between BMI and anteversion error in the surface registration group. Hohmann et al. (2011) reported that one of the most important factors to avoid high systemic errors is precise acquisition of the bony landmarks. Ybinger et al. (2007) also reported that the thickness of the soft tissue overlying bony landmarks influenced the inclination and the anteversion values. In contrast, Lass et al. (2014) found no statistically significant difference in accuracy of acetabular component position in relation to a patient’s BMI. However, they claimed that the main reason that BMI was not affecting the accuracy of navigation was exact acquisition of the bony landmarks with a sharp metal pointer.

Given the above, it seems reasonable to assume that the most important factor in determination of APP is the thickness of the soft tissue overlying bony landmarks affecting their precise registration, but it seems this does not affect our EMN system.

We also confirmed the second hypothesis and proved that the duration of the surgery was unaffected by the navigation system, which is in contrast to other published studies where the duration of surgery was considerably longer due to navigation (Parratte and Argenson 2007, Najarian et al. 2009, Lass et al. 2014, Liu et al. 2015). Considering that longer surgeries are associated with the increased risk of infection (Cheng et al. 2017, Kong et al. 2017), this represents an important advantage over other navigation systems. We observed an even narrower range of duration of the surgery in the EHIP group, which is probably due to more reproducible and fluent surgical procedures allowed by the EMN support.

Since the EMN system with Navi-frame is applicable to every patient’s position, and every surgical approach in THA, the main limitation of our study was to focus on the cup position only. We did not consider the combined acetabular and femoral component anteversion, which could represent another factor in the prevention of impingement and possible implant dislocation. In our study, femoral components were of rectangular, tapered, cementless design with limited ability to adjust their anteversion, which is determined by the femoral canal. The main influence on the combined anteversion was the acetabular component anteversion as noted also by Goudie et al. (2015). Based on a large metanalysis, cup position seems to be important for hip instability (particularly large deviations from the average) as well as many other variables. The target zone is difficult to set because it is influenced by many other factors including the approach and individual anatomical variations of the spinopelvic region (Seagrave et al. 2017). Additionally, given that several primary outcomes were tested, possible multiplicity issues were not excluded, and further studies are necessary to additionally confirm the clinical significance of the EMN system.

Nevertheless, based on our results, we could conclude that EMN in THA appears to increase the accuracy and precision of the cup position and does not affect the surgical duration. Consequently, it might become a valuable tool to target patient-specific cup position determined by many individual anatomical factors and judged important for hip stability and longevity. The best property of the EMN is that it is a passive system, providing the surgeon with real-time information on implant position. This is especially important in difficult anatomical situations, without standard landmarks and in mini-invasive procedures (DiGioia et al. 2003) where small incisions compromise the visibility of different landmarks, which usually help a surgeon in cup placement with the freehand technique.

The authors would like to thank the staff of the Department of Radiology at their institution for their support when performing the postoperative CT scans. Additionally, the authors would like to thank the staff of the A1 department at their institution, including nurses and physiotherapists, for their help in fulfilling pre- and postoperative protocols. Special thanks go to Anže Mihelič, MD, who was the third surgeon performing surgeries for the study.
